# Supplementary material for: Cost-Effectiveness Analysis of Pitavastatin in Dyslipidemia: Vietnam Case
Source: Healthcare (Basel). 2025 Oct 1;13(19):2494. doi: 10.3390/healthcare13192494 (PMC12524106; doi:10.3390/healthcare13192494)
Supplement: Supplementary file 1 [file healthcare-13-02494-s001.zip › healthcare-3834707-supplementary.pdf]

Supplemental

2. Materials and Methods

2.4.3. Utility

Table S1. Utility weights of patients achieving LDL-C targets.

Patient group	Baseline utility weight	Reference
Male (60–69 years)	0.8400	[1]
Female (60–69 years)	0.8110	[1]
Patients achieving LDL-C targets (in model)	0.8255	Calculated as the average utility of male and female patients

Table S2. Utility decrement associated with cardiovascular complications and the utility of patients not achieving the LDL-C target.

Patient group at risk of complications	Utility decrement	Reference
Myocardial infarction	-0.1185	[1]
Stroke	-0.1323	[1]
Revascularization	-0.1020	[1]
Average	-0.1176	Calculation
Utility of Patients not achieving the LDL-C target	0.7079	Calculation

3. Result

3.1. Cost, QALY

Table S3. Total cost of each event over a 14-year time horizon.

Branch	Category	Unit price (VND)	Quantity	Sum	Total cost (VND)
Patients treated with Pitavastatin are not achieving the LDL-C target	Pitavastatin 2 mg	13,500	56	756,000	94,582,600
	Pitavastatin 4 mg	18,500	28	518,000	
	Healthcare service	109,200	3	327,600	
	Pitavasatin 4mg (9-month maintenance)	18,500	5,026	92,981,000	
Patients using Pitavastatin 4 mg are achieving the LDL-C target	Pitavastatin 2 mg	13,500	56	756,000	94,582,600
	Pitavastatin 4 mg	18,500	28	518,000	
	Healthcare service	109,200	3	327,600	
	Pitavasatin 4mg (9-month maintenance)	18,500	5,026	92,981,000	

Patients using Pitavastatin 2 mg are achieving the target	Pitavastatin 2 mg	13,500	56	756,000	69,203,400
	Pitavastatin 2 mg (1-year maintenance)	13,500	28	378,000	
	Healthcare service	109,200	2	218,400	
	Pitavastatin 2 mg (9-month maintenance)	13,500	5,026	67,851,000	
Patients using Atorvastatin are not achieving the LDL-C target	Atorvastatin 10 mg	15,941	56	892,696	81,786,110
	Atorvastatin 20 mg	15,941	28	446,348	
	Healthcare service	109,200	3	327,600	
	Atorvastatin 20 mg (9-month maintenance)	15,941	5,026	80,119,466	
Patients using Atorvastatin 20 mg are achieving the LDL-C target	Atorvastatin 10 mg	15,941	56	892,696	81,786,110
	Atorvastatin 20 mg	15,941	28	446,348	
	Healthcare service	109,200	3	327,600	
	Atorvastatin 20 mg (9-month maintenance)	15,941	5,026	80,119,466	
Patients using Atorvastatin 10 mg are achieving the LDL-C target	Atorvastatin 10 mg	15,941	56	892,696	81,676,910
	Atorvastatin 10 mg (1-year maintenance)	15,941	28	446,348	
	Healthcare service	109,200	2	218,400	
	Atorvastatin 10 mg (9-month maintenance)	15,941	5,026	80,119,466	
Patients using Rosuvastatin are not achieving the LDL-C target	Rosuvastatin 5 mg	8,978	56	502,768	74,381,230
	Rosuvastatin 10 mg	14,553	28	407,484	
	Healthcare service	109,200	3	327,600	
	Rosuvastatin 10 mg (9-month maintenance)	14,553	5,026	73,143,378	
Patients using Rosuvastatin 10 mg are achieving the LDL-C target	Rosuvastatin 5 mg	8,978	56	502,768	74,381,230
	Rosuvastatin 10 mg	14,553	28	407,484	
	Healthcare service	109,200	3	327,600	
	Rosuvastatin 10 mg (9-month maintenance)	14,553	5,026	73,143,378	
Patients using Rosuvastatin 5 mg are achieving the LDL-C target	Rosuvastatin 5 mg	8,978	56	502,768	46,095,980
	Rosuvastatin 5 mg (1-year maintenance)	8,978	28	251,384	
	Healthcare service	109,200	2	218,400	
	Rosuvastatin 5 mg (9-month maintenance)	8,978	5,026	45,123,428	

References

1. Mould-Quevedo, J.F.; Gutiérrez-Ardila, M.V.; Ordóñez Molina, J.E.; Pinsky, B.; Vargas Zea, N. Cost-Effectiveness Analysis of Atorvastatin versus Rosuvastatin in Primary and Secondary Cardiovascular Prevention Populations in Brazil and Columbia. *Value Health Reg Issues* **2014**, *5*, 48-57. doi: 10.1016/j.vhri.2014.07.007
